# Ketamine-Induced Apoptosis in Normal Human Urothelial Cells: A Direct, N-Methyl-d-Aspartate Receptor–Independent Pathway Characterized by Mitochondrial Stress

**DOI:** 10.1016/j.ajpath.2015.12.014

**Published:** 2016-05

**Authors:** Simon C. Baker, Saqib Shabir, Nikolaos T. Georgopoulos, Jennifer Southgate

**Affiliations:** ∗ Jack Birch Unit of Molecular Carcinogenesis, Department of Biology, University of York, York, United Kingdom; † Department of Biological Sciences, School of Applied Sciences, University of Huddersfield, Huddersfield, United Kingdom

## Abstract

Recreational abuse of ketamine has been associated with the emergence of a new bladder pain syndrome, ketamine-induced cystitis, characterized by chronic inflammation and urothelial ulceration. We investigated the direct effects of ketamine on normal human urothelium maintained in organ culture or as finite cell lines *in vitro*. Exposure of urothelium to ketamine resulted in apoptosis, with cytochrome *c* release from mitochondria and significant subsequent caspase 9 and 3/7 activation. The anesthetic mode-of-action for ketamine is mediated primarily through *N*-methyl d-aspartate receptor (NMDAR) antagonism; however, normal (nonimmortalized) human urothelial cells were unresponsive to NMDAR agonists or antagonists, and no expression of NMDAR transcript was detected. Exposure to noncytotoxic concentrations of ketamine (≤1 mmol/L) induced rapid release of ATP, which activated purinergic P2Y receptors and stimulated the inositol trisphosphate receptor to provoke transient release of calcium from the endoplasmic reticulum into the cytosol. Ketamine concentrations >1 mmol/L were cytotoxic and provoked a larger-amplitude increase in cytosolic Ca^2+^ concentration that was unresolved. The sustained elevation in cytosolic Ca^2+^ concentration was associated with pathological mitochondrial oxygen consumption and ATP deficiency. Damage to the urinary barrier initiates bladder pain and, in ketamine-induced cystitis, loss of urothelium from large areas of the bladder wall is a reported feature. This study offers first evidence for a mechanism of direct toxicity of ketamine to urothelial cells by activating the intrinsic apoptotic pathway.

The phencyclidine derivative ketamine is an *N*-methyl-d-aspartate (NMDA) receptor antagonist,^1^ which is used as a rapid-onset, short-duration anesthetic and analgesic in clinical and veterinary practice. In the clinical setting, ketamine is particularly used as an anesthetic in pediatric and asthmatic cases, and for palliative care. Recreational use of ketamine, which has been increasing since the 1980s, is based around its phencyclidine-like effects, whereby it induces hallucinations, stimulates out-of-body experiences, and increases empathy and insight.^2^

The emergence of upper and lower urinary tract damage resulting from ketamine abuse was reported originally as case studies in 2007, where symptoms of urinary frequency/urgency, nocturia, hematuria, and suprapubic pain were accompanied by a thickened, contracted, and inflamed bladder.^3^, ^4^

The sloughing of the bladder epithelium (urothelium) observed by histology in ketamine cystitis patients is suggestive of a direct toxicity against the urothelium,^5^ but no mechanism has yet been established. Ketamine has been classified as an *N*-methyl d-aspartate receptor (NMDAR) antagonist,^1^ and *in vitro* studies with cortical neurons from rats^6^ and monkeys^7^ have identified the NMDAR as a mediator of ketamine-induced apoptosis. NMDAR subunits have been reported to be expressed in the urinary tract of rats,^8^ which suggests this as a potential mechanism in human urothelium. Our aim was to identify a mechanism for the direct toxicity of ketamine on normal (nonimmortalized) human urothelial (NHU) cells to enhance understanding of the clinical pathology.

## Materials and Methods

### Chemicals and Reagents

Racemic ketamine hydrochloride salt (without preservatives) was soluble in cell culture medium at 10 mmol/L and was 0.2 μm filter-sterilized before use. Unless specified otherwise, all chemicals were of analytical or tissue culture grade, as appropriate, and were obtained from Sigma-Aldrich Company Ltd. (Gillingham, UK).

Because of the high concentrations of ketamine used in this study (and recreationally), it was important to assess the effects of ketamine (0.1 to 10 mmol/L) on the osmolality of culture medium. No change in osmolality of complete keratinocyte serum-free medium (KSFMc) was observed at ketamine concentrations <5 mmol/L, and although slight increases were recorded at 5 mmol/L and greater, the concentrations used in this study did not exceed the normal osmolality range of serum (275 to 299 mOsm/kg).

### Urothelial Cell Isolation and Culture

Urothelium was collected with National Health Service Research Ethics Committee approval and required informed consent from urological procedures that excluded urothelial neoplasia. Finite (nonimmortal, serially passaged) NHU cell lines were established in KSFMc (Invitrogen, Paisley, UK) with cholera toxin, as described previously.^9^ For this study, cell lines derived from eight individuals were used up to passage 5.

Cell counts were performed using Trypan Blue exclusion to identify viable cells using an improved Neubauer Hemocytometer (SLS, Wilford, UK). To eliminate cyclic-adenosine monophosphate as a confounding factor, all experiments were performed in KSFMc without cholera toxin.

Organ cultures were established from human ureteric tissue and maintained in Dulbecco's modified Eagle's medium/RPMI 1640 medium (50:50 mix) supplemented with 5% fetal bovine serum. After 72 hours' exposure to 3 mmol/L ketamine, ureteric organ cultures from six donors were fixed in 10% formalin for 24 hours, dehydrated through graded alcohols, and embedded in paraffin wax for histological evaluation.

### Histological Evaluation of Organ Cultures

Dewaxed tissue sections (5 μm thick) were either stained with hematoxylin and eosin (following standard methods) or immunoperoxidase-labeled using the M30 Cytodeath antibody to cleaved cytokeratin 18 (Roche, Mannheim, Germany).

For immunoperoxidase labeling, blocking steps were included to neutralize endogenous peroxidase and avidin-binding activities. Heat-mediated antigen retrieval was performed by microwave boiling for 10 minutes in 10 mmol/L citric acid buffer (pH 6). After overnight incubation in primary antibody (diluted 1:100) at 4°C, slides were washed, incubated in biotinylated secondary antibodies and a streptavidin-biotin horseradish peroxidase complex (Dako Cytomation, Ely, UK), and visualized using a diaminobenzidine substrate reaction. Sections were counterstained with hematoxylin, dehydrated, and mounted in DPX (CellPath, Powys, UK).

### Quantification of Cell Number by Alamar Blue Assay

Alamar Blue (AbD Serotec, Kidlington, UK), diluted 1:10 with KSFMc, was added to cells grown in 96-well plates. After 4 hours' incubation at 37°C, the absorbance was measured at 570 and 630 nm. The reduction of the Alamar Blue dye is proportional to mitochondrial enzyme activity and can be used as a proxy for viable cell number.^10^

### Calcium Imaging

NHU cells were seeded at 5 × 10^4^ cells/cm^2^ onto collagen-coated (0.1 mg/mL rat-tail collagen; Becton Dickinson, Oxford, UK) glass coverslips and maintained for 24 hours. Before imaging, cultures were washed with HEPES-buffered saline solution (HBSS; 138 mmol/L NaCl, 5 mmol/L KCl, 0.3 mmol/L KH_2_PO_4_, 4 mmol/L NaHCO_3_, 0.3 mmol/L NaHPO_4_, 1 mmol/L MgCl_2_, 2 mmol/L CaCl_2_, and 10 mmol/L HEPES, pH 7.4) and then loaded with 5 μmol/L fluo-4(AM) and 5 μmol/L fura-red(AM) in HBSS with 0.02% pluronic acid for 25 minutes. Cultures were washed twice in HBSS, and the coverslips were placed in a perfusion chamber (Warner Instruments, supplied by Harvard Apparatus Ltd., Edenbridge, UK) on the stage of a Revolution XD spinning disk confocal microscope (Andor, Belfast, UK). The chamber was perfused with an automated pump (Scientifica, Uckfield, UK) at a flow rate of 1.5 mL/minute, and images were recorded at a rate of 1/second.

In some experiments, to differentiate exogenous from intrinsic calcium stores, cells were preincubated for 10 minutes in calcium-free HBSS (138 mmol/L NaCl, 5 mmol/L KCl, 0.3 mmol/L KH_2_PO_4_, 4 mmol/L NaHCO_3_, 0.3 mmol/L NaHPO_4_, 1 mmol/L MgCl_2_, 5 mmol/L EGTA, and 10 mmol/L HEPES, pH 7.4), and experiments were performed in the calcium-free buffer.

Changes in the intensity of the fura-red and fluo-4 fluorescent signal were measured in all of the cells in the field of view using Andor iQ software version 2.1 (Andor, Belfast, UK), which was used to record and export raw data over time. The data were imported in Origin 8.6 (Origin Labs, Microcal, Stoke Mandeville, UK), and the ratio of fluorescence was calculated to indicate the change in intracellular Ca^2+^ concentration.

### RT-PCR

RNA was extracted in Trizol reagent (Thermo Fisher Scientific, Loughborough, UK), any contaminating DNA was digested using a DNA-free kit (Thermo Fisher Scientific), and cDNA was synthesized using Oligo(dT) 12-18 primers (Invitrogen). RT-PCR was performed using SureStart Taq polymerase (Agilent Technologies LDA UK Ltd., Stockport, UK) and a thermal cycler (PCR Express; Hybaid, Thermo Fisher Scientific). With the following annealing temperatures, primers were designed to amplify GRIN1 (forward, 5′-GCATCCTCGGGCTGCAGCTC-3′ and reverse, 5′-AGCGGCCCGGTCTTCCAGAT-3′, 61°C), GRIN2A (forward, 5′-GTGGTCTATCAACGGGCAGT-3′ and reverse, 5′-AGGTGAGACGGTGCCATTAC-3′, 51°C), GRIN2B (forward, 5′-GATGGGAGCCCCTACGCCCA-3′ and reverse, 5′-CCACCGTGGGCTGCCTGAAG-3′, 60°C), GRIN2C (forward, 5′-CGACGCCAGCCACGTGAGTT-3′ and reverse, 5′-AGAGCACCTCGGCCTCCTCG-3′, 54°C, with 5% dimethyl sulfoxide added to the reaction), GRIN2D (forward, 5′-CCACCTTCCTGCAGCTGGGC-3′ and reverse, 5′-GAGCTGGGCACTGAGCACGG-3′, 61°C), glyceraldehyde-3-phosphate dehydrogenase (forward, 5′-ACCCAGAAGACTGTGGATGG-3′ and reverse, 5′-TTCTAGACGGCAGGTCAGGT-3′, 60°C), and UPK2 (forward, 5′-CTCCCGCAAGTAAGGAGGT-3′ and reverse, 5′-GAAGGATGGGGGAATTGTTA-3′, 58°C) transcripts. Reverse transcription negative controls were included in all experiments.

### Mitochondrial OCR Monitoring

The mitochondrial oxygen consumption rate (OCR) of NHU cells in the presence and absence of 3 mmol/L ketamine was assessed using an XFp Analyzer to perform an XF Cell Mitochondrial Stress Test, according to the manufacturer's instructions (Seahorse Bioscience, Copenhagen, Denmark), as detailed.^11^ Briefly, NHU cells were seeded at 1 × 10^4^ cells per well and cultured for 48 hours to near confluence, when cultures were exposed to medium containing 3 mmol/L ketamine and maintained for a further 48 hours. Before the assay, cultures were washed with, and then incubated in, XF Assay modified Dulbecco's modified Eagle's medium (unbuffered; Seahorse Bioscience) containing 5 mmol/L l-glutamine, 5 mmol/L sodium pyruvate, and 6 mmol/L glucose. The XFp Analyzer performed fluorimetric detection of oxygen consumption by sequentially adding and mixing 1 μmol/L oligomycin, 1 μmol/L carbonyl cyanide-4-(trifluoromethoxy)phenylhydrazone (FCCP), and 0.5 μmol/L antimycin A with 0.5 μmol/L rotenone at defined time points. Values were normalized to total cellular protein derived from a bicinchoninic acid assay (Pierce, supplied by Thermo Fisher Scientific), and mitochondrial respiration was quantified by normalization to OCR values in the presence of antimycin A and rotenone, as described elsewhere.^11^

### TMRM Staining and Flow Cytometry

NHU cell cultures were treated for 48 hours with either control KSFMc or 3 mmol/L ketamine in KSFMc, washed twice with KSFMc, and labeled with 200 nmol/L tetramethylrhodamine (TMRM) methyl ester (Molecular Probes; supplied by Thermo Fisher Scientific) in KSFMc for 30 minutes at 37°C. Live cultures were imaged immediately at fixed exposures using an Olympus B60 epifluorescent microscope (Southend-on-Sea, UK).

For flow cytometry, the same procedure was followed, but cells were first harvested with trypsin in versene before TMRM staining was performed in suspension. Staining was quantified using a CyAn ADP analyzer (Beckman Coulter Ltd, High Wycombe, UK), and data were summarized for three independent donor cell lines as the mean of median fluorescence from each donor, normalized to the no ketamine control.

### Cellular ATP Assay

Cellular ATP was quantified using the ENLITEN ATP assay, according to the manufacturer's instructions (Promega Ltd, Southampton, UK), and normalized using a bicinchoninic acid protein assay. Briefly, NHU cells were cultured to 80% confluence in 10 cm dishes before exposure to 3 mmol/L ketamine or medium only (KSFMc) for 48 hours. Cultures were harvested, lysed in 2% trichloroacetic acid, and assayed for activation of luciferase against an ATP standard curve in a luminescence plate reader (BMG Labtech Ltd., Aylesbury, UK).

### Western Blot Analysis

Whole cell lysates were obtained from cultures by *in situ* lysis in SDS electrophoresis sample buffer, containing 13 mmol/L dithiothreitol and 0.2% (v/v) protease inhibitors (Protease Inhibitor Cocktail set III; Calbiochem, supplied by Merck Chemicals Ltd., Beeston, UK), after two phosphate-buffered saline washes. Cells from at least three donors were used in all experiments. Mitochondrial and cytosolic fractions were prepared from all (adherent and detached) cells using the MIT1000 kit, according to the manufacturer's instructions (Merck Chemicals, Ltd.). Lysates were sonicated and microcentrifuged, and the protein concentration was determined by Bradford colorimetric assay. Protein samples (20 to 40 μg) were resolved by electrophoresis of NuPAGE gels using the Novex system (Invitrogen) and electrotransferred to polyvinylidene difluoride membranes (Merck Millipore). Membranes were blocked for 1 hour in Odyssey blocking buffer (Li-Cor Biosciences UK Ltd., Cambridge, UK), incubated with titrated primary antibody overnight at 4°C. Primary antibodies were raised against serine 473 phosphorylated-Akt (Cell Signaling Technology Europe, Leiden, the Netherlands; 9271, rabbit; 1:1000 dilution), threonine 202/tyrosine 204 phosphorylated-ERK1/2 (Cell Signaling; 9101, rabbit; 1:1000 dilution), serine 9 phosphorylated glycogen synthase kinase (GSK) 3β (Abcam plc, Cambridge, UK; ab30619, rabbit; 1:500 dilution), Bcl-2 (Merck Millipore; clone 100, mouse; 1:1000 dilution), cytochrome *c* (Santa Cruz, supplied by Insight Biotechnology Ltd., Wembley, UK; H-104, rabbit; 0.4 μg/mL), caspase 9 (Cell Signaling; 9502, rabbit; 1:1000 dilution), caspase 3 (Cell Signaling; 9662, rabbit; 1:1000 dilution) and cleaved-poly (ADP ribose) polymerase (Cell Signaling; 5625, rabbit; 1:1000 dilution). Densitometry was normalized using the intensity of housekeeping proteins β-actin (Sigma-Aldrich; clone AC15, mouse; 1:10,000 dilution) or glyceraldehyde-3-phosphate dehydrogenase (Merck Millipore; clone 6C5, mouse; 1:2000 dilution). Membranes were labeled with the appropriate IRDye-conjugated secondary antibody and visualized using an Odyssey Infrared imager (Li-Cor).

### Caspase 3/7 Assay

NHU cell cultures were seeded at 1.5 × 10^5^ cells/well in 96-well plates (Corning Primaria, Fisher Scientific UK Ltd.), and 24 hours after seeding were treated with ketamine in replicates of six. After 72 hours, apoptosis was assessed with the Sensolyte active caspase 3/7 kit used according to the manufacturer's instructions (Anaspec; supplied by Cambridge Bioscience, Cambridge, UK). Briefly, cells were lysed and any active capase-3/7 cleaved a quencher from the AFC (7-amido-4-trifluoromethylcoumarin) fluorophore to generate bright blue fluorescence. Caspase activity–associated fluorescence was measured on a PolarStar Optima plate reader (BMG LabTech) at excitation and emission wavelengths of 355 and 492 nm, respectively.

### Statistical Analysis

Means were compared using the appropriate statistical tests in Graphpad InStat statistical software version 3.05 (GraphPad, La Jolla, CA), and repeated-measures analysis of variance was performed in SPSS version 20 (IBM, Portsmouth, UK). *P* < 0.05 was considered significant.

## Results

### Direct Exposure of Urothelium to Ketamine

**Figure 1:**
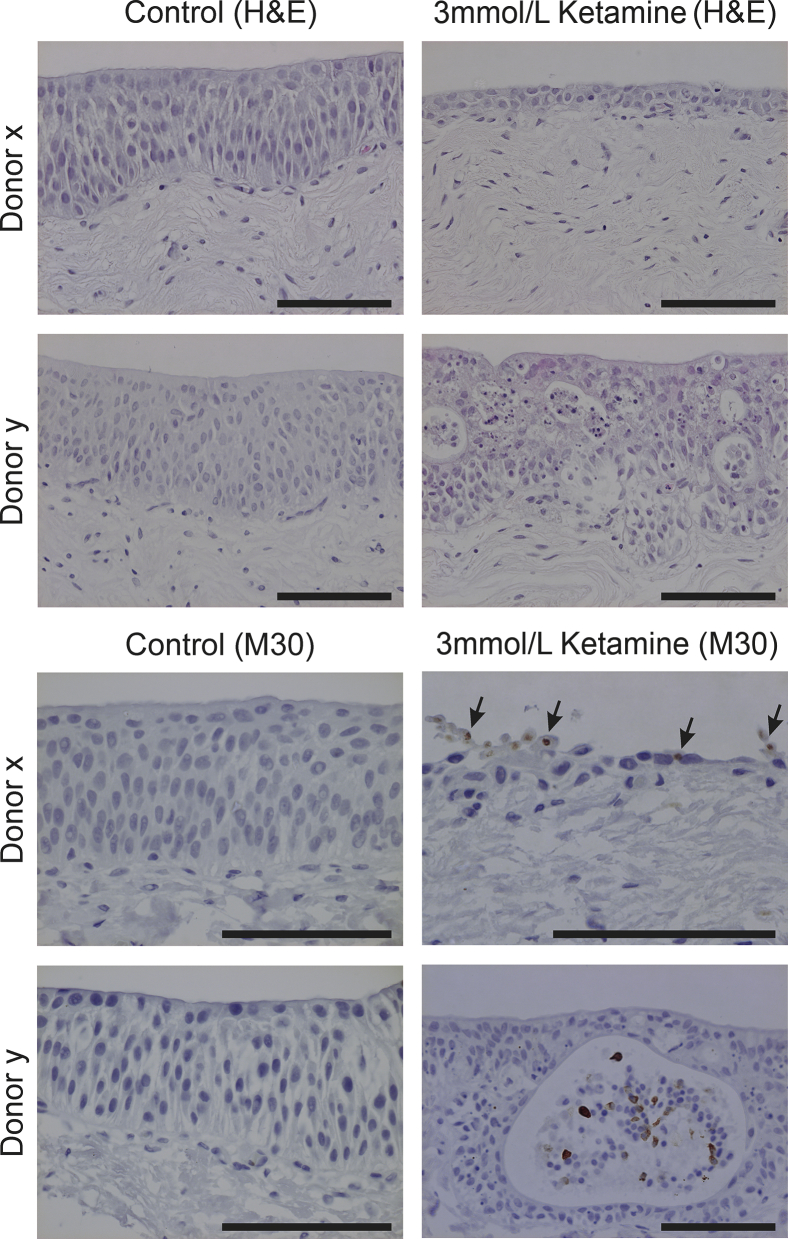
Histological assessment of human ureteric organ cultures after 72 hours' exposure to 3 mmol/L ketamine showed thinning of the epithelium (shown in the images of donor x) and clear signs of apoptosis, including pyknotic nuclei and karyorrhexis (shown in the images of donor y). Immunoreactivity with a cleaved cytokeratin 18 antibody (M30 Cytodeath) was observed in cells being lost from the epithelium (**arrows**). *n* = 6 donors with two representative examples shown. Scale bar = 100 μm. H&E, hematoxylin and eosin.

Organ cultures of human ureters from six donors were used to examine the direct effect of ketamine exposure to the urothelium (Figure 1). Hematoxylin and eosin staining of ureters after 72 hours' exposure to 3 mmol/L ketamine showed thinning of the epithelium throughout the organ culture in five of six donors (Figure 1), with pyknotic nuclei and karyorrhexis evident as cells were lost from the urothelium (Figure 1). Immunolabelling of organ cultures with a cleaved-cytokeratin 18 antibody showed immunoreactivity in cells being lost from the epithelium, indicating the importance of apoptosis (Figure 1).

**Figure 2:**
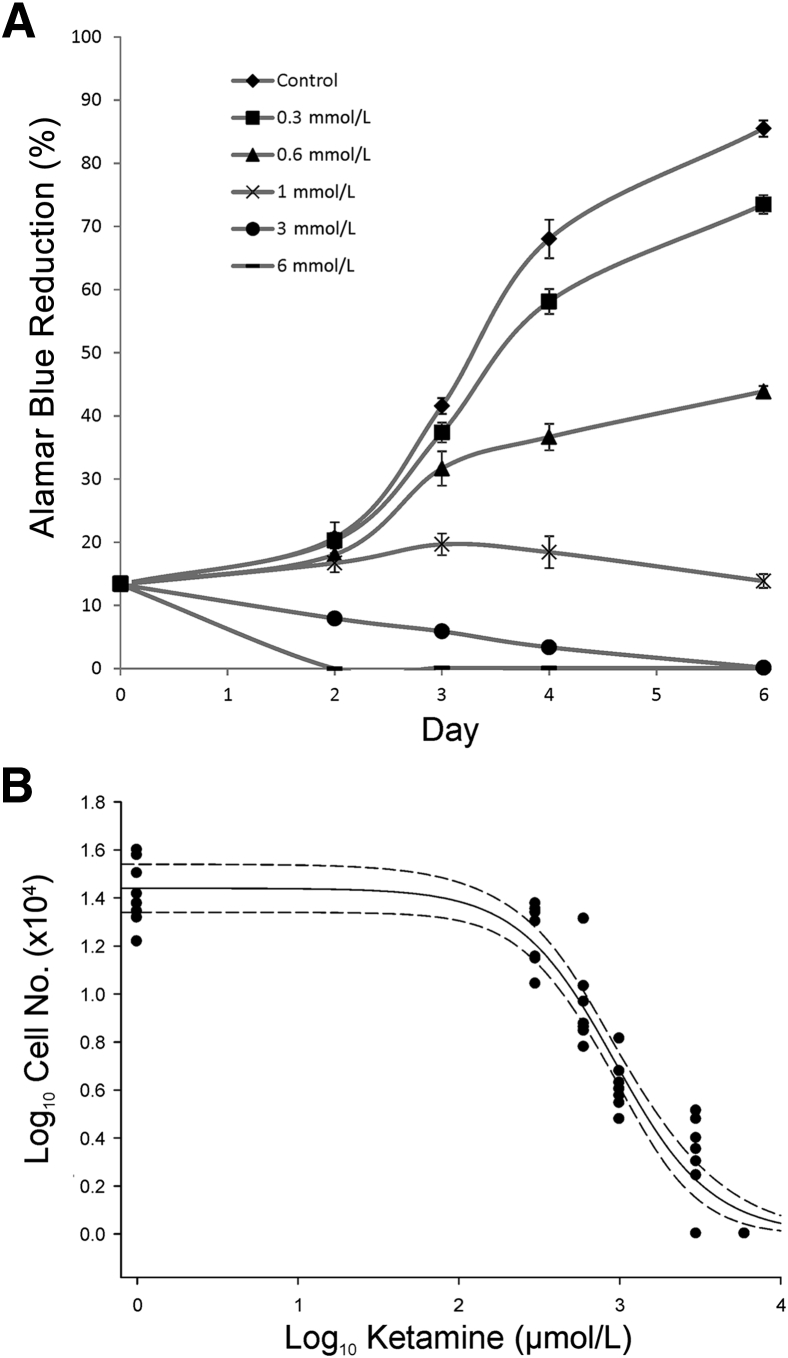
Effects of ketamine on population growth in cultures of normal (nonimmortalized) human urothelial (NHU) cells. **A:** Concentrations of ketamine between 0.3 and 6 mmol/L were assessed during a 6-day time course by Alamar Blue reduction assay. This graph is representative of repeats in three independent donor cell lines. **B:** Counts from two donor cell lines after 96 hours of exposure to ketamine indicated a half maximal inhibitory concentration (IC_50_) for NHU cells of 0.93 mmol/L. The IC_50_ was calculated using a sigmoidal fit and is illustrated as a **solid black line**; the 95% CI is shown as **dashed lines**. Error bars represent SD (**A**). *n* = 6 (**A**); *n* = 4 replicates per donor (**B**).

To examine the mechanisms of ketamine action further, nonimmortalized NHU cell cultures were exposed to ketamine during a 6-day time course. Ketamine induced a reproducible concentration-dependent effect, being growth inhibitory at ≥0.3 mmol/L, cytostatic at 1 mmol/L, and toxic at >1 mmol/L (Figure 2A). Counts of proliferating NHU cell cultures after 4 days of exposure found the half-maximal inhibitory concentration for ketamine to be 0.93 mmol/L (Figure 2B). On the basis of these data, the cytostatic/half-maximal inhibitory concentration of 1 mmol/L ketamine was used in comparison with a cytotoxic dose of 3 mmol/L for all further studies.

**Figure 3:**
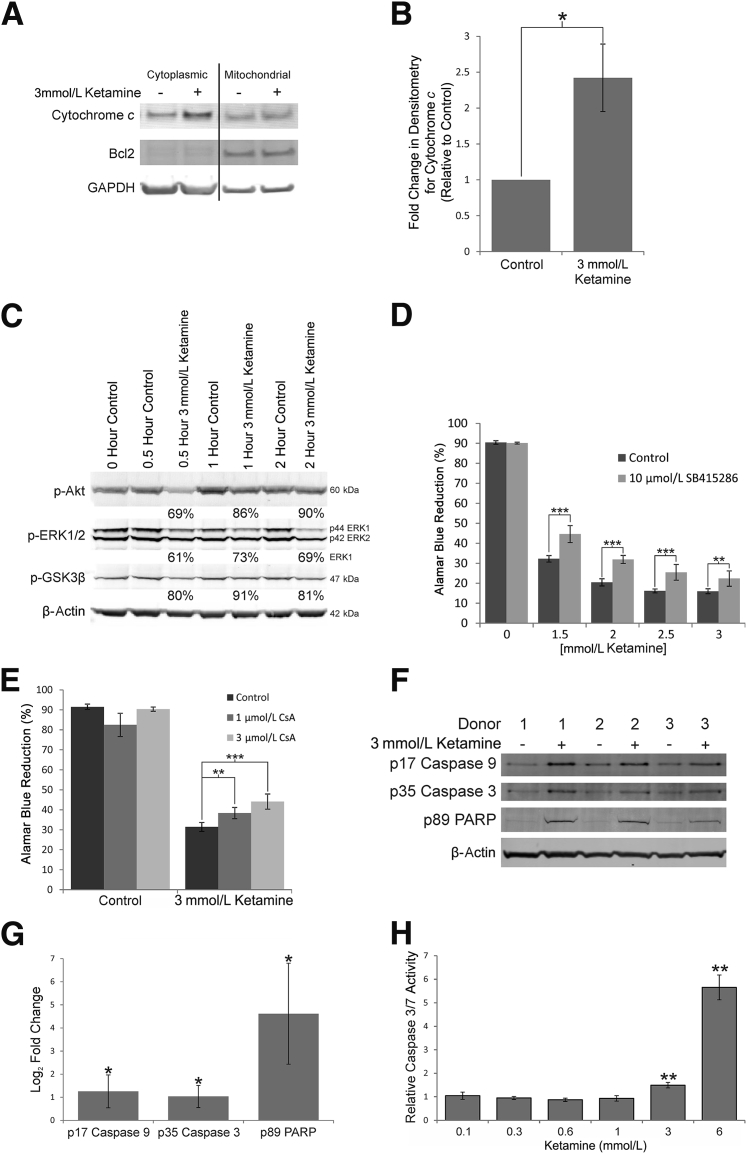
Induction of apoptosis in normal (nonimmortalized) human urothelial (NHU) cell cultures by ketamine. **A:** Western blot analysis of NHU cells separated into cytoplasmic and mitochondrial fractions showed elevated cytochrome *c* in the cytosolic fractions of cultures treated with 3 mmol/L ketamine for 24 hours. Loading controls were Bcl2 for the mitochondrial fraction and glyceraldehyde-3-phosphate dehydrogenase (GAPDH) for the cytoplasmic fraction. **B:** Densitometry analysis showed a significant mean 2.4-fold change in cytoplasmic cytochrome *c* content in three independent NHU cell lines after 24 hours' exposure to 3 mmol/L ketamine. **C:** Western blot analysis of phospho-Akt (active form), phospho-ERK1/2 (active form), and S9 phospho-glycogen synthase kinase (GSK) 3β (inactive form) showed early depletion of these forms of the kinases in response to 3 mmol/L ketamine. Abundance quantified by densitometry is shown as a percentage of control cells for each time point, normalized to β-actin (combined data). **D:** Inhibition of GSK3β by SB415286 in ketamine-exposed NHU cells was capable of a slight, but significant, inhibition of toxicity, as assessed by Alamar Blue reduction. **E:** Inhibition of the mitochondrial permeability transition pore with cyclosporin A (CsA) in ketamine-exposed NHU cells was capable of a small, but significant, inhibition of toxicity, as assessed by Alamar Blue reduction. **F:** Western blot analysis of caspase 9, caspase 3, and cleaved poly (ADP-ribose) polymerase (PARP) in NHU cells after 72 hours' exposure to 3 mmol/L ketamine. **G:** The three markers of apoptosis were all significantly increased by more than twofold in densitometry, which was normalized to β-actin. **H:** Caspase 3/7 activity was assessed in lysates from NHU cell cultures exposed to 0.1 to 6 mmol/L ketamine and normalized to baseline caspase activity in untreated cells. Significant increases in caspase activity were observed after exposure to 3 and 6 mmol/L ketamine. Error bars represent SD (**B**, **D**, **E**, **G**, and **H**). *n* = 4 donors (**C**); *n* = 6 (**D**, **E**, and **H**); *n* = 3 donors (**B** and **G**). ^∗^*P* < 0.05, ^∗∗^*P* < 0.01, and ^∗∗∗^*P* < 0.001.

Adherent and detached NHU cells exposed to 3 mmol/L ketamine were collectively lysed, and cytoplasmic- and mitochondrial-enriched fractions were prepared to study the localization of the apoptotic mediator cytochrome *c*. The cytoplasmic fractions of ketamine-treated cells showed a significant mean 2.4-fold increase in cytochrome *c* when compared with time-matched controls after 24 hours of exposure (repeated-measures analysis of variance *P* < 0.05, *n* = 3) (Figure 3, A and B). Exposure to 3 mmol/L ketamine caused a rapid reduction in phosphorylation of Akt (serine 473) and ERK1/2 (threonine 202/tyrosine 204), with a consequential reduction of inhibition of GSK3β activity (Figure 3C). The serine 9 phosphorylated form of GSK3β is inactive, and 3 mmol/L ketamine reduced phosphorylation (Figure 3C), indicating increased GSK3β activity that could contribute to mitochondrial permeability transition pore activation.^12^ Inhibition of GSK3β with SB415286 showed a small, but significant, alleviation of the >1 mmol/L ketamine-induced toxicity at 48 hours (analysis of variance with Tukey-Kramer multiple comparisons post test *P* < 0.01, *n* = 6) (Figure 3D). Furthermore, inhibition of the mitochondrial permeability transition pore with cyclosporin A showed a significant concentration-dependent inhibition of toxicity at 48 hours (analysis of variance with Tukey-Kramer multiple comparisons post test *P* < 0.01, *n* = 6) (Figure 3E). Cotreatment of NHU cells with 3 mmol/L ketamine and 3 μmol/L cyclosporin A led to a 1.4-fold increase in cell viability at 48 hours, compared with cells treated with ketamine alone (*n* = 12 measurements of cells from two independent donors).

Exposure to 3 mmol/L ketamine for 72 hours induced significant increases in the abundance of caspase 9 (mean, 2.6-fold), caspase 3 (mean, 2.1-fold), and cleaved poly (ADP-ribose) polymerase (mean, 48.5-fold), as assessed by densitometry of Western blots (Figure 3, F and G). A caspase 3/7 activity assay recorded significant increases in caspase activity detected after 72 hours with ketamine at concentrations ≥3 mmol/L (Figure 3H).

### NMDAR Expression by Human Urothelium

**Figure 4:**
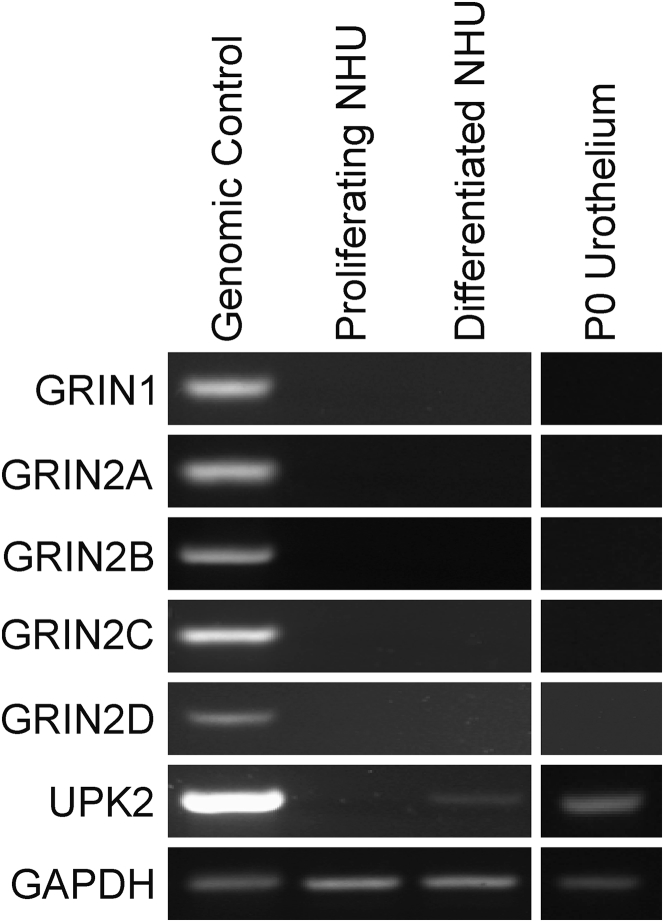
RT-PCR of NMDA receptor transcripts (GRIN isoforms). No expression of GRIN isoforms was detected in normal (nonimmortalized) human urothelial (NHU) cells under either proliferating or differentiated cell culture conditions. Furthermore, no GRIN expression was detected in freshly isolated urothelium after its separation from the underlying stroma. Representative images of one donor (proliferating and differentiated NHU) and one donor P0 urothelium are shown. Uroplakin 2 (UPK2) transcript expression was used to confirm urothelial differentiation, and glyceraldehyde-3-phosphate dehydrogenase (GAPDH) was included as an internal housekeeping control to confirm RNA integrity. In addition, no products were detected in reverse transcriptase–negative cDNA controls that were generated using each RNA preparation.

Ketamine is an NMDAR antagonist; however, pretreatment of NHU cells with a combination of 1 mmol/L d-serine and 100 μmol/L NMDA was unable to inhibit calcium transients or block ketamine-induced toxicity (data not shown). The potent, specific NMDAR antagonist MK-801 (alias dizocilpine, which has an NMDAR dissociation constant 100-fold greater than ketamine^13^) did not trigger any toxicity or calcium transients at concentrations ≤100 μmol/L (data not shown). The lack of any effects triggered by specific agonists/antagonists of the NMDAR led to evaluation of the expression of NMDAR (GRIN) isoform transcripts by proliferating and differentiated NHU cell cultures; no expression of GRIN1, 2A, 2B, 2C, or 2D was detected (Figure 4). To ensure that lack of GRIN transcripts was not an artifact of culturing urothelial cells, transcript expression was analyzed in freshly isolated urothelium, which was removed from the underlying stroma to prevent contamination with other cell types. No expression of GRIN isoform transcripts was detected in freshly isolated *in situ* human urothelium (Figure 4).

### The Role of Calcium Signaling in the NHU Cell Toxicity of Ketamine

**Figure 5:**
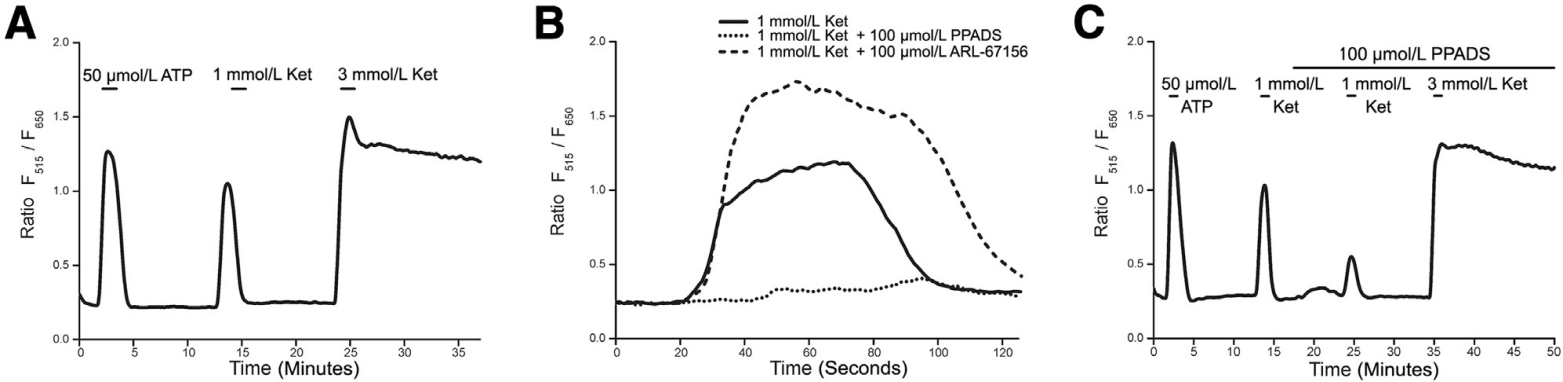
Treatment of normal (nonimmortalized) human urothelial (NHU) cells with ketamine (Ket) resulted in elevation of cytosolic [Ca^2+^]. **A:** The addition of 1 mmol/L ketamine (noncytotoxic) to a culture of NHU cells produced a transient increase in cytosolic [Ca^2+^], whereas exposure to 3 mmol/L ketamine (cytotoxic) induced a higher [Ca^2+^] in the cytoplasm, which did not return to a normal baseline. **B:** The role of purinergic receptors in mediating the urothelial response to 1 mmol/L ketamine was investigated by preincubating cultures for 10 minutes in 100 μmol/L pyridoxal-phosphate-6-azophenyl-2′,4′-disulfonate (PPADS; purinergic receptor inhibitor) or ARL-67156 (ecto-ATPase inhibitor). PPADS (dotted line) almost completely blocked the calcium transient observed after addition of 1 mmol/L ketamine to NHU cells, whereas ARL-67156 (dashed line) increased both the amplitude and duration of the transient. **C:** Preincubating cultures in PPADS was not sufficient to inhibit the [Ca^2+^] increase triggered by 3 mmol/L ketamine. All images are traces representative of triplicate experiments.

The cytostatic concentration of 1 mmol/L ketamine was seen to elicit an increase in cytosolic calcium concentration in 95% of NHU cells in culture; this transient was of comparable intensity to that stimulated by 50 μmol/L ATP, and all cells that responded to ATP were responsive to ketamine (Figure 5A). The amplitude of the calcium transient in response to ketamine was concentration-dependent, and the duration was associated with the observed toxicity. In NHU cells exposed to ≤1 mmol/L ketamine for 1 minute, the calcium signal returned to baseline after an average of 90 seconds, whereas 3 mmol/L ketamine for 1 minute invoked a sustained elevation in the cytosolic calcium concentration that remained unresolved after 10 minutes (Figure 5A).

The role of ATP in driving the observed elevations of cytosolic calcium concentration was elucidated by cotreatment of 1 mmol/L ketamine with an ecto-ATPase inhibitor 6-*N*,*N*-diethyl-d-β-γ-dibromomethylene-ATP (ARL-67156), which significantly enhanced the amplitude and duration of the calcium transient (Figure 5B). By contrast, pretreatment and coadministration of the purinergic receptor inhibitor pyridoxal-phosphate-6-azophenyl-2′,4′-disulfonate (PPADS) with 1 mmol/L ketamine almost completely abrogated the calcium transient (Figure 5B). Although these results demonstrated ATP was critical to the calcium transients stimulated by 1 mmol/L ketamine, it was not possible with PPADS to inhibit the nonresolving elevation of cytosolic calcium triggered by 3 mmol/L ketamine (Figure 5C).

**Figure 6:**
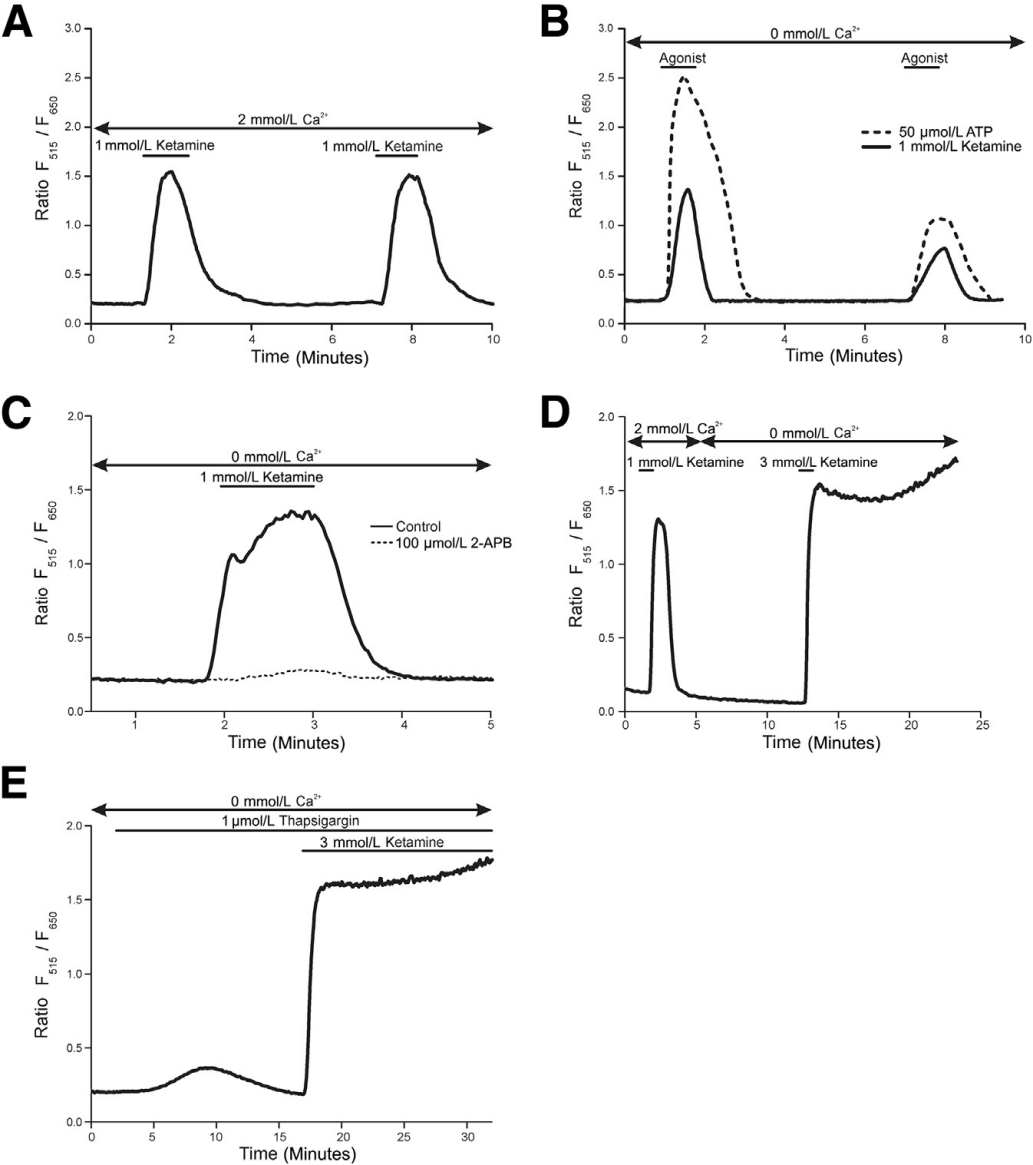
Normal (nonimmortalized) human urothelial (NHU) cell cystolic [Ca^2+^] was elevated by the release of stores from the endoplasmic reticulum in response to ketamine exposure. **A:** The repeated administration of 1 mmol/L ketamine to NHU cells in the presence of 2 mmol/L extracellular [Ca^2+^] resulted in repeated transient increases in cytosolic [Ca^2+^], with no sign of reduced intensity in subsequent transients. **B:** The source of the calcium transient was elucidated by performing repeated challenges of urothelial cells with 1 mmol/L ketamine (solid line) and 20 μmol/L ATP (dashed line) in the absence of extracellular calcium. An initial treatment with either ketamine or ATP generated a calcium transient showing that, in both cases, calcium was released from internal stores. A second stimulation triggered a significantly attenuated transient in response to either ketamine or ATP, indicating incomplete refilling of the internal stores. **C:** Pretreatment of urothelial cell cultures with 100 μmol/L 2-APB produced complete inhibition (dashed line) of the 1 mmol/L ketamine-induced calcium release (solid line). **D:** The source of the sustained [Ca^2+^] elevation was confirmed as internal by stimulating the cells with 3 mmol/L ketamine in the absence of exogenous calcium. **E:** Thapsigargin was used to inhibit SERCA pumps and effectively empty the ER by allowing Ca^2+^ ions to leak out. The 3 mmol/L ketamine-induced elevation in cytosolic [Ca^2+^] persisted. Images are traces representative of triplicate experiments.

To understand the source and mechanism of ketamine-induced calcium release, cultures were challenged with 1 mmol/L ketamine, which, in the presence of 2 mmol/L (near-physiological) extracellular calcium, produced repeated calcium transients of similar intensity (Figure 6A). Although an initial transient elevation in cytosolic calcium concentration stimulated by 1 mmol/L ketamine was also observed in the absence of extracellular calcium, a second challenge produced a smaller calcium response than the first because of incomplete refilling of calcium stores in the endoplasmic reticulum (ER) (Figure 6B). This was similar to the effect observed on repeated ATP stimulation in the absence of exogenous calcium and demonstrated that the transient elevations in cytosolic calcium concentration were derived from intracellular stores (Figure 6B). When cultures were pretreated with the inositol trisphosphate (IP_3_) receptor inhibitor 2-aminoethoxydiphenyl borate (2-APB), in the absence of extracellular calcium, the 1 mmol/L ketamine-stimulated transient elevation in cytosolic calcium concentration was lost, revealing the source of calcium to be the ER (Figure 6C). It was not possible to inhibit the nonresolving elevation of cytosolic calcium concentration triggered by 3 mmol/L ketamine using 2-APB (data not shown).

The prolonged elevation in cytosolic calcium concentration induced by 3 mmol/L ketamine in 2 mmol/L extracellular calcium (Figure 5A) was reproduced when cultures were stimulated by 3 mmol/L ketamine in the absence of extracellular calcium, showing this process was also driven entirely by the release of intracellular calcium stores (Figure 6D). Using the sarco/endoplasmic reticulum Ca^2+^ ATPase (SERCA) inhibitor/ER calcium ATPase inhibitor, thapsigargin, to prevent refilling of the ER (and therefore induce its emptying through calcium ion leakage), it was observed that the 3 mmol/L ketamine-induced elevation of cytosolic calcium concentration persisted, suggesting this calcium was not derived from a thapsigargin-sensitive region of the ER (Figure 6E).

PPADS, 2-APB, and BAPTA-AM [1,2-bis(o-aminophenoxy)ethane-N,N,N′,N′-tetraacetic acid-acetoxymethyl ester] could not be titrated to inhibit ketamine toxicity, as assessed by Alamar Blue reduction (data not shown).

### Mitochondrial Physiology

**Figure 7:**
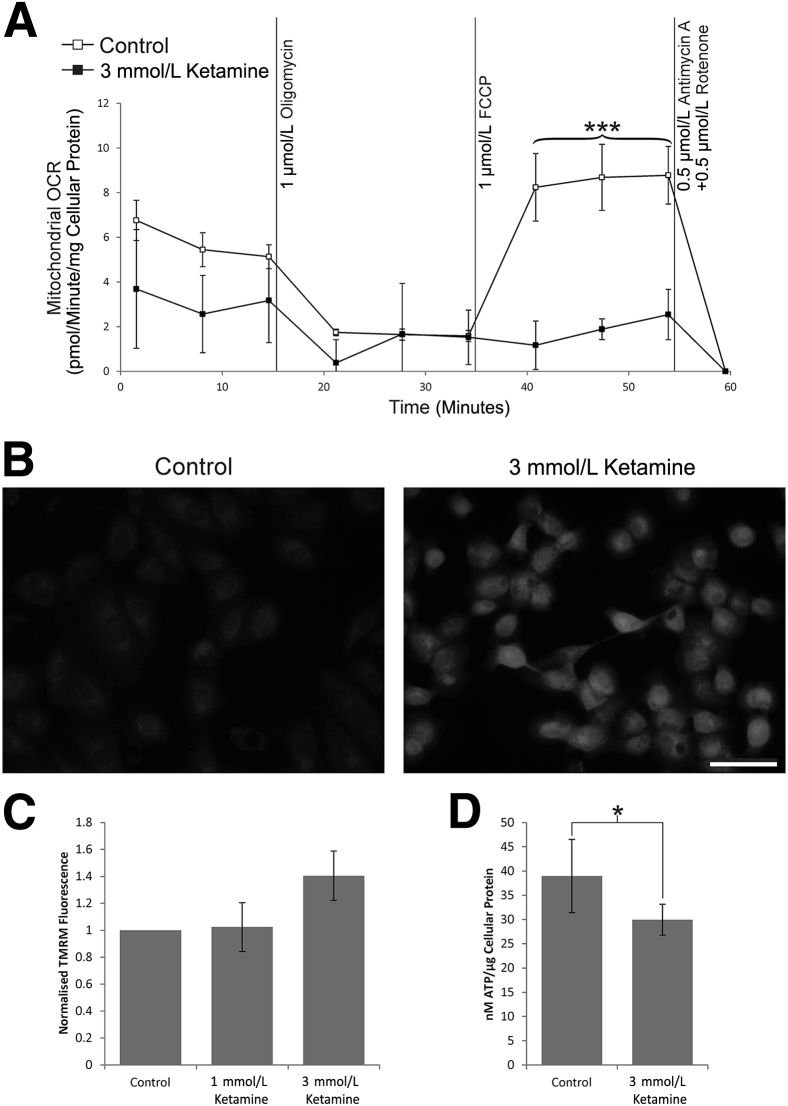
Ketamine exposure causes mitochondrial stress in normal (nonimmortalized) human urothelial (NHU) cells. **A:** NHU cells exposed to 3 mmol/L ketamine for 48 hours had a significantly lower resting mitochondrial oxygen consumption rate (OCR) than controls (54.4%; Mann-Whitney test *P* < 0.001). The proportion of OCR devoted to physiology other than ATP generation remained the same, whereas the spare mitochondrial capacity in control cells was far greater (4.6-fold; Mann-Whitney test). Data are the means of experiments performed in duplicate on three independent cell lines. **B:** NHU cells labeled with tetramethylrhodamine (TMRM) showed that 3 mmol/L ketamine exposure led to elevated mitochondrial membrane potential (images are representative of experiments performed in cells from three donors). **C:** Results in **B** were confirmed quantitatively by flow cytometry (mean of three independent donor cell lines given). **D:** Cellular ATP was significantly reduced to 76.8% of control after 3 mmol/L ketamine exposure for 48 hours. Data are the means of duplicate measurements in three independent donor cell lines. Error bars represent SD (**A**, **C**, and **D**). ^∗^*P* < 0.05, ^∗∗∗^*P* < 0.001. Scale bar = 50 μm (**B**). FCCP, carbonyl cyanide-4-(trifluoromethoxy)phenylhydrazone.

NHU cells exposed to 3 mmol/L ketamine for 48 hours had a significantly lower resting mitochondrial OCR than controls (mean, 54.4%; Mann-Whitney test *P* < 0.001) (Figure 7A). The decrease in mitochondrial OCR in response to inhibition of ATP synthase (1 μmol/L oligomycin) indicated the proportion of OCR that related to the generation of ATP. Subtracting the mitochondrial OCR after ATP synthase inhibition, from the resting value, suggested control cells used more oxygen to generate ATP (mean, 2.1-fold; Mann-Whitney test *P* < 0.003) (Figure 7A). Addition of the ionophore and uncoupling agent FCCP (1 μmol/L) equalized the proton gradient across the inner mitochondrial membrane and defined the maximum OCR for NHU cell mitochondria (Figure 7A). The maximum mitochondrial OCR in control cells was significantly greater than for ketamine-exposed cells (mean, 4.6-fold; Mann-Whitney *U* test *P* < 0.001).

TMRM labeling of NHU cells showed greater intensity in cultures exposed to 3 mmol/L ketamine for 48 hours, indicating that mitochondria became hyperpolarized (Figure 7, B and C). A combination of low mitochondrial OCR with high mitochondrial membrane potential suggested ketamine-exposed cells consumed ATP and used ATP synthase to maintain the proton gradient. Cellular ATP assays showed that ATP was significantly depleted after 48 hours of exposure to 3 mmol/L ketamine (76.8% of control cells; *t*-test with Welch correction *P* < 0.05) (Figure 7D).

## Discussion

The data reported herein suggest that ketamine-induced toxicity to urothelial cells is associated with prolonged elevation of cytosolic calcium concentration triggered by high concentrations of ketamine (>1 mmol/L). High and sustained intracellular calcium ion concentrations can lead to mitochondrial stress and ultimately cytochrome *c* release, which causes caspase activation and apoptosis.^14^ The role of apoptosis in ketamine-induced cystitis was supported using human urothelial organ cultures that showed pyknotic nuclei, karyorrhexis, and immunoreactivity with a cleaved-cytokeratin 18 antibody.

**Figure 8:**
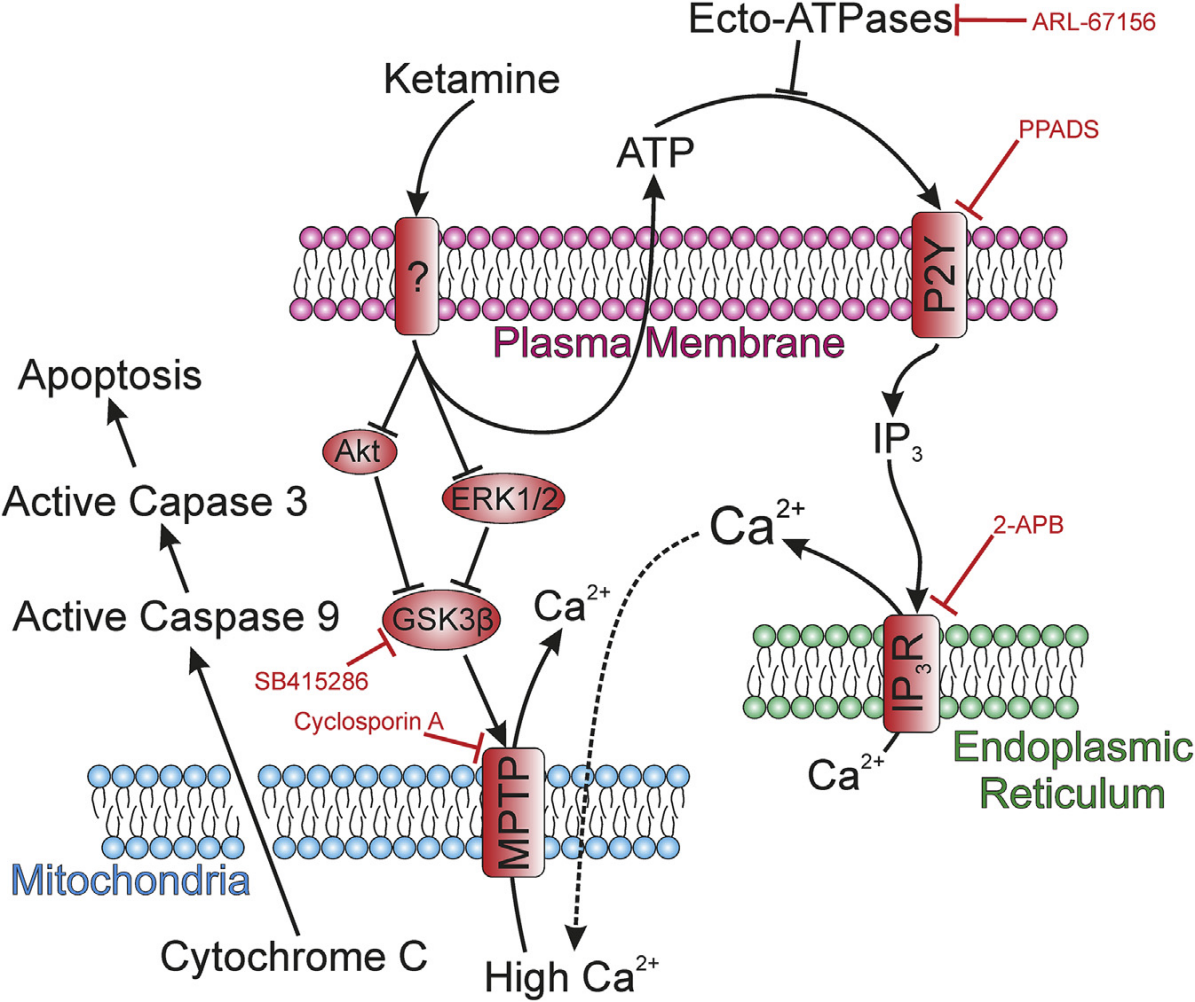
Schematic diagram summarizing the proposed mechanism of ketamine-induced cytotoxicity downstream of initial receptor activation/inhibition and illustrating how the experiments performed in this study support the proposed mode-of-action. Ketamine exposure leads to ATP release from the cells (the ATP signal could be enhanced by preventing its breakdown with ARL-67156), which binds to P_2_Y receptors [that were effectively inhibited by pyridoxal-phosphate-6-azophenyl-2′,4′-disulfonate (PPADS)]. Activation of P_2_Y receptors releases inositol trisphosphate (IP_3_), which binds IP_3_ receptor on the endoplasmic reticulum [inhibited using 2-aminoethoxydiphenyl borate (2-APB)] and causes release of stored Ca^2+^ into the cytoplasm. Prolonged elevation of cytoplasmic [Ca^2+^] triggers further Ca^2+^ release from mitochondria, probably via the activated mitochondrial permeability transition pore (MPTP). Ketamine exposure triggers a reduction in the phosphorylated/active forms of Akt and ERK, promoting an increase in glycogen synthase kinase (GSK) 3β activity (which can be inhibited by SB415286). Acting in concert, GSK3β activity and sustained elevation of cytosolic [Ca^2+^] activate the MPTP (which can be inhibited by cyclosporin A), leading to mitochondrial depolarization, release of cytochrome *c*, and caspase-mediated apoptosis.

Our study demonstrates, for the first time, that ketamine induces an increase in cytosolic calcium concentration in urothelial cells via release of calcium from internal stores rather than influx from the extracellular milieu, suggesting disruption of organelle function. The observation that ketamine-induced calcium transients were blocked with 2-APB confirmed that the ketamine-induced calcium response was because of an IP_3_-mediated calcium release from the ER. Exacerbation of calcium transients by inhibiting ecto-ATPases (with ARL-67156) and abrogation after P2Y inhibition (by PPADS) suggest that ketamine stimulates ATP release from urothelial cells, which then binds purinergic receptors on the cell surface, allowing IP_3_ to trigger calcium release from the ER. The purinergic receptors involved in this process are almost certain to be of the P2Y family (known to be functional in human urothelium^15^) because P2X receptors act as channels, allowing extracellular calcium into the cytoplasm, and the ketamine-induced calcium release observed persisted in the absence of extracellular calcium. This proposed P2Y-mediated action of ketamine (Figure 8) is supported by similar findings in a rat microglial cell line,^16^ which is the only other report of intracellular calcium transients triggered by ketamine.

The source of calcium that delivers the sustained elevation observed during 3 mmol/L ketamine exposure remains elusive; however, others have reported thapsigargin-insensitive stores of calcium in the Golgi and high concentrations of calcium ions in some regions of the ER.^17^ In addition, it has been reported that thapsigargin-insensitive calcium stores cannot be mobilized by IP_3_-release (as triggered by 1 mmol/L ketamine), which might suggest the extra calcium released by 3 mmol/L ketamine is accessed via a different mechanism.^18^ Intense TMRM labeling of ketamine-exposed cells suggests the mitochondria in most adherent cells retain their membrane potential and, therefore, are unlikely to be the source of the global increase in cytosolic calcium concentration.

The mitochondria of ketamine-exposed urothelial cells displayed extremely low OCR, with only slight inhibition by oligomycin (suggesting little ATP generation) and no detectable increase after FCCP addition (demonstrating a lack of spare capacity for ATP generation). These data support the concept that the electron transport chain is largely inactive in the mitochondria of ketamine-exposed cells and, combined with intense TMRM labeling (showing the inner mitochondrial membranes were hyperpolarized), suggest that the mitochondria had become ATP consumers. By running ATP synthase in reverse, mitochondria can maintain membrane potential and survive stressors that inhibit functioning of the electron transport chain.^19^ The depletion of cellular ATP in ketamine-exposed cells provides additional support for their inhibited bioenergetics. At a certain threshold, individual mitochondria will run out of ATP, membrane potential will no longer be maintained by ATP synthase, and the mitochondrial permeability transition pore will form, releasing cytochrome *c* and activating caspases (including 9 and 3) to initiate apoptosis. The involvement of mitochondria in caspase activation in response to ketamine by human lymphocytes and neuronal cells was previously reported,^20^ and is an established mechanism of toxicity for other xenobiotics in the urothelium.^21^

Studies of cultured rat^6^ and monkey^7^ cortical neurons identified the NMDAR as the mediator of ketamine-induced apoptosis in these cells. By contrast, our study found no expression of GRIN transcripts and no response to NMDAR agonists or more specific antagonists by human urothelial cells *in vitro* or *in situ*. Notably, previous reports of NMDAR expression in human and rat urinary tract tested homogenized urothelial/stromal tissue together.^8^ In a study of human lymphocytes and neuronal cells, the low stereospecificity of ketamine-induced apoptosis was taken as evidence against the involvement of NMDAR, which has a higher affinity for S-ketamine,^20^ suggesting that the significance of non-NMDAR targets of ketamine may not be confined to the urothelium. The initial receptor responsible for stimulating the ATP release remains elusive and is particularly difficult to identify because of the recognized receptor-binding promiscuity of ketamine.

We previously reported nerve hyperplasia present in ketamine cystitis patients, which might play a role in pain generation.^5^ Herein, we report urothelial ATP release in response to ketamine exposure, which may be another factor in the extreme pain experienced, because ATP acting on afferent nerves in the bladder wall is a widely accepted cause of pelvic pain.^22^

Recreational ketamine consumption tends to be orders of magnitude higher (54% of users have taken >1 g in a session^23^) than in the low (50 mg) oral dose clinical studies on which predicted urine concentrations are based.^24^ A young adult male taking 1 g of ketamine could expect 85% of the drug to be excreted in the urine within 24 hours,^24^ and taking into account the average voiding rate of 6 × 300 mL per day,^25^ a urine concentration in excess of 1 mmol/L is theoretically possible, suggesting the scale of *in vitro* toxicity reported herein (and by others in cancer cell lines^26^) is relevant clinically. Formal support would require accurate quantification of ketamine/metabolite concentrations in the urine of symptomatic recreational users. Nevertheless, our biological observation of a sustained, calcium-induced, intrinsic apoptotic pathway–driven response that may drive extensive urothelial tissue damage, resulting in chronic inflammation, provides valuable clues to explain the reported pathology associated with ketamine cystitis. Moreover, in light of the recent drive by the pharmaceutical sector to produce new-generation ketamine derivatives for clinical use as novel antidepressants,^27^ this model of bladder cells will be valuable in ensuring that any urological effects are identified and closely monitored.
